# Haploinsufficient *Rock1*^+/−^ and *Rock2*^+/−^ Mice Are Not Protected from Cardiac Inflammation and Postinflammatory Fibrosis in Experimental Autoimmune Myocarditis

**DOI:** 10.3390/cells9030700

**Published:** 2020-03-12

**Authors:** Karolina Tkacz, Filip Rolski, Marcin Czepiel, Edyta Działo, Maciej Siedlar, Urs Eriksson, Gabriela Kania, Przemysław Błyszczuk

**Affiliations:** 1 Department of Clinical Immunology, Jagiellonian University Medical College, 30-663 Cracow, Poland; karolina.tkacz@student.uj.edu.pl (K.T.); Filip.rolski@uj.edu.pl (F.R.); marcinczepiel@gmail.com (M.C.); edyta.dzialo@doctoral.uj.edu.pl (E.D.); misiedla@cyf-kr.edu.pl (M.S.); 2 Cardioimmunology, Center for Molecular Cardiology, University of Zurich, 8952 Schlieren, Switzerland; urs.eriksson@uzh.ch; 3 Center of Experimental Rheumatology, Department of Rheumatology, University Hospital Zurich, 8952 Schlieren, Switzerland; gabriela.kania@uzh.ch

**Keywords:** rho-associated protein kinase, ROCK1, ROCK2, experimental autoimmune myocarditis, cardiac fibroblasts, inflammatory cells, TGF-β, cardiac fibrosis

## Abstract

Progressive cardiac fibrosis is a common cause of heart failure. Rho-associated, coiled-coil-containing protein kinases (ROCKs) have been shown to enhance fibrotic processes in the heart and in other organs. In this study, using wild-type, *Rock1*^+/−^ and *Rock2*^+/−^ haploinsufficient mice and mouse model of experimental autoimmune myocarditis (EAM) we addressed the role of ROCK1 and ROCK2 in development of myocarditis and postinflammatory fibrosis. We found that myocarditis severity was comparable in wild-type, *Rock1*^+/−^ and *Rock2*^+/−^ mice at day 21 of EAM. During the acute stage of the disease, hearts of *Rock1*^+/−^ mice showed unaffected numbers of CD11b^+^CD36^+^ macrophages, CD11b^+^CD36^–^Ly6G^hi^Ly6c^hi^ neutrophils, CD11b^+^CD36^–^Ly6G^–^Ly6c^hi^ inflammatory monocytes, CD11b^+^CD36^–^Ly6G^–^Ly6c^–^ monocytes, CD11b^+^SiglecF^+^ eosinophils, CD11b^+^CD11c^+^ inflammatory dendritic cells and type I collagen-producing fibroblasts. Isolated *Rock1*^+/−^ cardiac fibroblasts treated with transforming growth factor-beta (TGF-β) showed attenuated Smad2 and extracellular signal-regulated kinase (Erk) phosphorylations that were associated with impaired upregulation of smooth muscle actin alpha (αSMA) protein. In contrast to cardiac fibroblasts, expanded *Rock1*^+/−^ heart inflammatory myeloid cells showed unaffected Smad2 activation but enhanced Erk phosphorylation following TGF-β treatment. *Rock1*^+/−^ inflammatory cells responded to TGF-β by a reduced transcriptional profibrotic response and failed to upregulate αSMA and fibronectin at the protein levels. Unexpectedly, in the EAM model wild-type, *Rock1*^+/−^ and *Rock2*^+/−^ mice developed a similar extent of cardiac fibrosis at day 40. In addition, hearts of the wild-type and *Rock1*^+/−^ mice showed comparable levels of cardiac vimentin, periostin and αSMA. In conclusion, despite the fact that ROCK1 regulates TGF-β-dependent profibrotic response, neither ROCK1 nor ROCK2 is critically involved in the development of postinflammatory fibrosis in the EAM model.

## 1. Introduction

Cardiac fibrosis is a pathogenic condition of the cardiac muscle leading to heart failure. In the process of fibrogenesis, physiological structure of the myocardium is replaced by an exaggerated accumulation of stromal cells and extracellular matrix (ECM) components in the tissue [1]. Pathological remodelling of the cardiac tissue can result in stiffening and enlargement of the ventricles and aberrant conduction of electrical impulses leading to systolic and/or diastolic dysfunctions and arrhythmias. Cardiac fibrosis develops following major ischemic events in the heart, mainly as a reparative process, but it occurs also in a number of nonischemic cardiac disorders as a response to increased blood pressure or following systemic or heart-specific inflammation [2].

Inflammation of the cardiac muscle is referred to as myocarditis. Cardiotropic infections or tissue damage followed by activation of heart-specific autoimmunity are common causes of myocarditis in humans [3]. In 14%–52% of biopsy-proven cases, myocarditis progresses to a dilated cardiomyopathy phenotype that is characterized by extensive fibrosis, ventricular dilation and heart failure [4]. Animal models represent useful tools to study pathogenesis of myocarditis, and mouse models of experimental autoimmune myocarditis (EAM) reflect key aspects of the human disease [5]. Immunization of susceptible mice with α-myosin heavy chain alpha (α-MyHC) together with complete Freund’s adjuvant represents the most commonly used noninfectious model of myocarditis. In this EAM model, the disease is mediated by autoreactive CD4^+^ T cells and is manifested by a massive infiltration of inflammatory cells, mainly of the myeloid lineage. Resolution of the inflammation is followed by the progressive accumulation of fibrotic tissue in the myocardium, ventricular dilatation and systolic dysfunction in many mice [6].

At the molecular level, fibrogenesis is triggered by a number of profibrotic inputs, which activate a complex signalling network. In cardiac fibrosis, transforming growth factor β (TGF-β) is recognised as a master profibrotic cytokine. Constitutive overexpression of TGF-β was shown to induce interstitial cardiac fibrosis and cardiac hypertrophy in transgenic mice [7]. In the EAM model, inactivation of TGF-β with blocking antibodies during acute inflammatory phase successfully prevented development of postinflammatory cardiac fibrosis [8]. TGF-β triggers a canonical response involving Smad proteins that become phosphorylated and translocated into the nucleus, where they act as transcription factors. At the same time, TGF-β activates a number of Smad-independent signalling pathways, such as phosphatidylinositol-3-kinase/Akt, Wnt or mitogen-activated protein kinases MEK/extracellular signal-regulated kinase (Erk) among others [9,10].

RhoA-Rho-associated, coiled-coil-containing protein kinase (ROCK) axis represents another example of Smad-independent TGF-β signalling [11]. RhoA belongs to the Rho GTP-ase family and functions as a switch protein between the active GTP-bound (RhoA-GTP) and inactive GDP-bound (RhoA-GDP) forms [12]. ROCKs exist in two isoforms, ROCK1 and ROCK2, and they represent the main effector proteins of the RhoA pathway. These serine–threonine kinases have been shown to regulate actin filament architecture, cell motility, autophagy, differentiation and apoptosis in many cell types [13].

A growing body of evidence indicates that ROCKs play an important role in cardiac fibrosis in ischemic and in nonischemic heart failures [14]. In the mouse model of myocardial infarction, *Rock1*^−/−^ and *Rock1*^+/−^ mice showed reduced cardiac fibrosis [15,16]. Similar protective effects were observed in animals treated with pharmacological ROCK inhibitors fasudil or ZYZ-168 [17,18,19,20]. The profibrotic role of ROCK1 and ROCK2 has been also demonstrated in models of hypertension induced with angiotensin II infusion or by transverse aortic constriction [16,18,21,22,23]. Furthermore, *Rock1* deficiency prevented development of cardiac fibrosis in mice with cardiomyocyte-restricted overexpression of Gαq [14]. In line with these data, overexpression of an active ROCK1 in cardiomyocytes induced fibrotic cardiomyopathy, which was further augmented by angiotensin II infusion or by Gαq [14,24]. Recent data showed that disruption of both *Rock1* and *Rock2* genes in cardiomyocytes reduced cardiac fibrosis during ageing [25]. Profibrotic ROCK activity, however, is not only restricted to cardiomyocytes. ROCK2 expression in activated fibroblasts was shown to promote cardiac fibrosis in mice receiving continuous angiotensin II infusions [26]. All these data clearly point to an active role of ROCKs in cardiac fibrogenesis in noninflammatory condition. The role of ROCK1 and ROCK2 in inflammatory heart disease remains, however, unknown.

## 2. Methods

### 2.1. Mice

*Rock1*^+/−^ and *Rock2*^+/−^ haploinsufficient mice were kindly provided by Dr. J.K. Liao. Reporter mice expressing enhanced green fluorescent protein (EGFP) under collagen type I promoter (Coll-EGFP) were described previously [27]. Transgenic mice were back-crossed for at least 10 generations on BALB/c background. Mice were kept under standard laboratory conditions: 12/12h light/dark cycle, room temperature 20–22 °C, humidity 45%–55% with access to food and water ad libitum. All experiments were performed in accordance with Swiss and Polish law and were approved by local authorities (license number ZH49/2009 and ZH194/2012 for Switzerland and 67/2015, 207/2017 and 372A/2020 for Poland). Animal experiment followed the Guide for the Care and Use of Laboratory Animals, published by the US National Institutes of Health [28].

### 2.2. EAM Induction

EAM was induced in 6–8-week-old mice by subcutaneous injection of 200 µg of α-MyHC_614-634_ peptide (Ac-RSLKLMATLFSTYASADR-OH, Caslo, Lyngby, Denmark) emulsified 1:1 with complete Freund’s adjuvant (CFA, BD Difco, Franklin Lakes, NJ, USA) at day 0 and 7. At the end of the experiment, mice were euthanized by anaesthetic overdose administered intraperitoneally.

### 2.3. Cell Cultures

Inflammatory myeloid cells (referred to earlier as prominin-1^+^/CD133^+^ progenitors) were obtained as described previously [8,29] with minor modifications. Briefly, myocarditis-positive hearts at day 17–21 of EAM were perfused and dissected into pieces and incubated in the Liberase solution (Roche, Basel, Switzerland) for 45–60 min at 37 ˚C. Cellular suspensions were filtered through 70 µm and 40 µm cell strainers and plated in the culture medium containing Iscove’s modified Dulbecco’s medium (Corning, New York, NY, USA) supplemented with 20% foetal bovine serum (FBS, Gibco, Waltham, WA, USA), penicillin–streptomycin (1:100, Gibco) and β-mercaptoethanol (1:1000, Sigma-Aldrich, Saint Louis, USA). Cardiac fibroblasts were isolated from hearts of healthy mice and cultured in the Dulbecco’s Modification of Eagle’s Medium supplemented with 10% FBS (Gibco), penicillin–streptomycin (1:100, Gibco) and β-mercaptoethanol (1:1000, Sigma). For experiments, cells at the first or second passage were used. Both types of cells were treated with 10 ng/mL TGF-β (PeproTech, London, UK) for 30 min, 1 h, 6 h, 24 h and 3 days before harvesting. Control cells were cultured without TGF-β.

### 2.4. Flow Cytometry

Single-cell suspensions from perfused mouse hearts were prepared by mechanical dissection and enzymatic digestion with the Liberase solution (Roche) for 45–60 min at 37 ˚C, followed by cell separation using 70 µm and 40 µm cell strainers. Cells were suspended in the flow cytometry buffer (2% FBS, 1mM EDTA in phosphate-buffered saline (PBS)) and treated with Fc receptor blocker (anti-CD16/32 antibody, 1:100, clone number 93, BioLegend, San Diego, USA) for 15 min at 4 ˚C. Next, cells were washed with flow cytometry buffer and incubated for 30 min on ice with fluorochrome-conjugated antibodies: anti-CD11b-PE (1:600, M1/70, Invitrogen, Carlsbad, CA, USA), anti-Ly6C-BV (1:300, HK1.4, BioLegend), anti-Ly6G-PECy7 (1:300, 1A8-Ly6g, Invitrogen), anti-SiglecF-Alexa700 (1:300, 1RNM44N, Invitrogen,), anti-CD36-APC (1:300, HM36, Invitrogen), anti-CD11c-PerCp (1:300, N418, BioLegend), anti-CD45-BV (1:200, 30-F11, Invitrogen) and anti-CD64-PE (1:300, X54-5/7.1, BioLegend). Cell viability was assessed using propidium iodide (Invitrogen). Samples were collected using BD FACSCanto™ II analyzer (BD Biosciences, San Jose, CA, USA), and the data were analysed with the FlowJo software (Tree Star, FlowJo X 10.0.7., Ashland, AS, USA). To determine absolute cell count, the Precision Count Beads (BioLegend) were used. Number of total cells per heart were calculated in reference to total volume of samples.

### 2.5. Histology and Immunohistochemistry

Mouse heart tissues were fixed in 4% formalin and embedded in paraffin. Conventional Hematoxylin/Eosin and Masson’s trichrome staining were used to assess cardiac inflammation and fibrosis, respectively. Myocarditis severity was assessed on Hematoxylin/Eosin sections at day 21 of EAM and graded from 0 to 4, as described before [29]. The Masson’s trichrome staining was used to evaluate fibrotic area in cardiac tissue at day 40 of EAM. Immunohistochemistry was performed as described previously [30] using the following antibodies: anti-mouse αSMA (clone E184, Abcam, Cambridge, UK), anti-mouse periostin (Abcam) and anti-mouse vimentin (Abcam). Immunopositive areas were quantified as a percentage in relation to total area of heart section using the ImageJ software (Version 1.52a, NIH, Bethesda, MA, USA) and custom-made plug-ins.

### 2.6. Immunoblotting

Inflammatory myeloid cells and cardiac fibroblasts were lysed in RIPA buffer supplemented with protease and phosphatase inhibitors (Thermo Fisher Scientific, Waltham, WA, USA). Immunoblotting procedure was performed as described previously [31] using the following antibodies: anti-Smad2/3 (1:1000, D43B4, Cell Signaling Technology), anti-phospho-Smad2/3 (1:1000, S465/467, Cell Signaling Technology), anti-MEK1/2 (1:1000, D1A5, Cell Signaling Technology), anti-phospho-MEK1/2 (1:1000, S217/221, Cell Signaling Technology), anti-Erk1/2 (1:1000, 137F5, Cell Signaling Technology), anti-phospho-Erk1/2 (1:1000, D13.14.4E, Cell Signaling Technology), anti-αSMA (1:1000, 1A4, BioLegend), anti-fibronectin (1:1000, polyclonal, Abcam), anti-ROCK1 (1:700, 46/ROCK-I, BD Transduction Laboratories) antibodies and anti-GAPDH (1:5000, 14C10, Cell Signaling Technology). The protein signal was detected using the Western Blotting Substrate (Thermo Fisher Scientific) and imaged with the ChemiDoc instrument (Bio-Rad, ChemiDoc Imaging System, Hercules, CA, USA). Results were analyzed with the ImageJ software (Version 1.52a, NIH, Bethesda, MA, USA). Protein abundance was normalized to GAPDH levels.

### 2.7. Quantitative RT-PCR

RNAs were extracted using the QIAzol Lysis Reagent (Qiagen, Hilden, Germany) according to manufacturer’s recommendations. mRNAs were reverse transcribed using the NG dART RT Kit (EurX). Quantitative real-time PCR was performed using the SYBR Green PCR Master Mix (EurX) and oligonucleotides complementary to transcripts of the analysed genes (Table S1) using the Quant Studio 7 Real-Time PCR system (Applied Biosystems, QuantStudio 7 Flex, Foster City, CA, USA). Transcript levels of *Gapdh* were used as endogenous reference, and the relative gene expression was calculated using the 2^−∆∆Ct^ method.

### 2.8. Immunocytochemistry

Cells were cultured and differentiated on gelatine-coated coverslips. Cells were fixed with 4% paraformaldehyde for 15 min at room temperature followed by blocking and permeabilization with 5% FBS and 0.1% Triton in PBS for 30 min at room temperature. Staining was performed using the primary anti-αSMA antibody (1:200, 1A4, BioLegend, San Diego, CA, USA) at room temperature, followed by the secondary AlexaFluor555 goat anti-mouse (1:1000, Invitrogen) antibody and phalloidin (1:1000, Thermo Fisher Scientific) at room temperature. Nuclei were stained with Hoechst 33342 (1:1000, Santa Cruz Biotechnology). Coverslips were mounted using the ProLong™ Diamond Antifade Mountant (Thermofisher) and examined under fluorescent microscope Olympus IX70 (Olympus, Shinjuku, Japan).

### 2.9. Statistics

Where relevant, the data were analysed by unpaired, two-tailed Student’s *t*-test and one-way ANOVA, followed by the Fisher’s Least Significant Difference post-hoc test for normally distributed data or Kruskal–Wallis test for nonparametric data. The Grubbs test was performed to identify outliers. Differences were considered statistically significant for p<0.05. All analyses were performed with GraphPad Prism 6 software (San Diego, CA, USA) and values are expressed as mean with standard error of the mean (SEM).

## 3. Results

### 3.1. Rock1^+/−^ and Rock2^+/−^ Haploinsufficient Mice Develop Unaffected Myocarditis

In order to induce EAM, *Rock1*^+/−^ and *Rock2*^+/−^ mice were back-crossed onto BALB/c genetic background. *Rock1*^+/−^ and *Rock2*^+/−^ haploinsufficient BALB/c mice were fertile and viable with no obvious phenotypic abnormalities. However, crossing *Rock1*^+/−^ BALB/c mice resulted in markedly underrepresented *Rock1*^−/−^ homozygotes. Out of 252 litters, only two (instead of 63 expected) showed *Rock1*^−/−^ genotype (Supplementary Figure S1). Crossing of *Rock2*^+/−^ BALB/c resulted, instead, in no viable *Rock2*^−/−^ offspring (Supplementary Figure S1).

**Figure 1:**
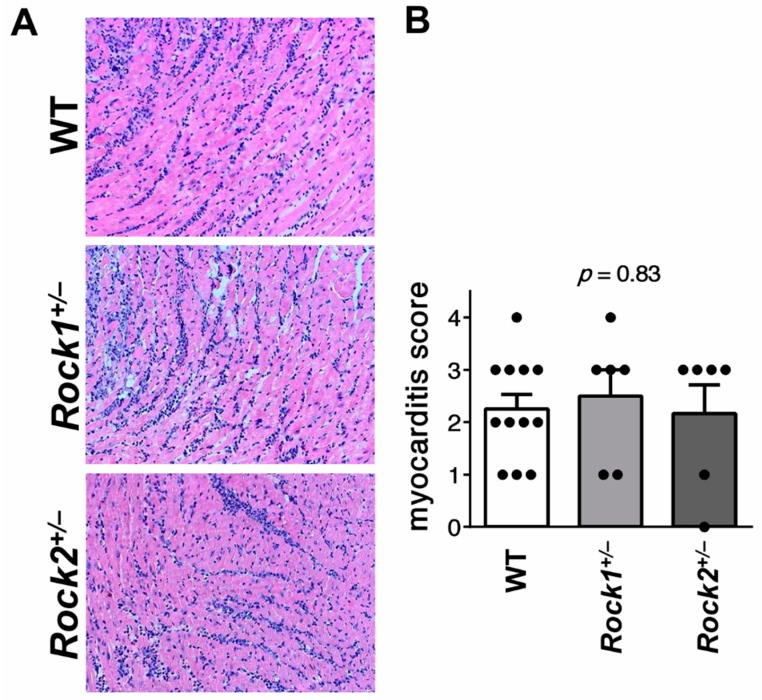
Unaffected myocarditis in *Rock1*^+/−^ and *Rock2*^+/−^ mice Panel (**A**) shows representative hematoxylin and eosin staining of heart tissue sections obtained from wild-type (n = 12), *Rock1*^+/−^ (n = 6) and *Rock2*^+/−^ (n = 6) mice at day 21 of experimental autoimmune myocarditis (EAM). Magnification x100. Quantification of myocarditis severity by using a semiquantitative 0-4 scale is shown in (**B**). Each dot represents data for one mouse and bars present mean value ± SEM. *p*-value was calculated with the Kruskal–Wallis test.

Using the haploinsufficient mice, we addressed whether ROCK1 or ROCK2 was implicated in the development of CD4^+^ T cell-mediated myocarditis. EAM was induced by α-MyHC/CFA immunization. Histological analysis of heart sections at day 21 of EAM showed that both *Rock1*^+/−^ and *Rock2*^+/−^ mice developed myocarditis with comparable severity to control wild-type mice (Figure 1).

**Figure 2:**
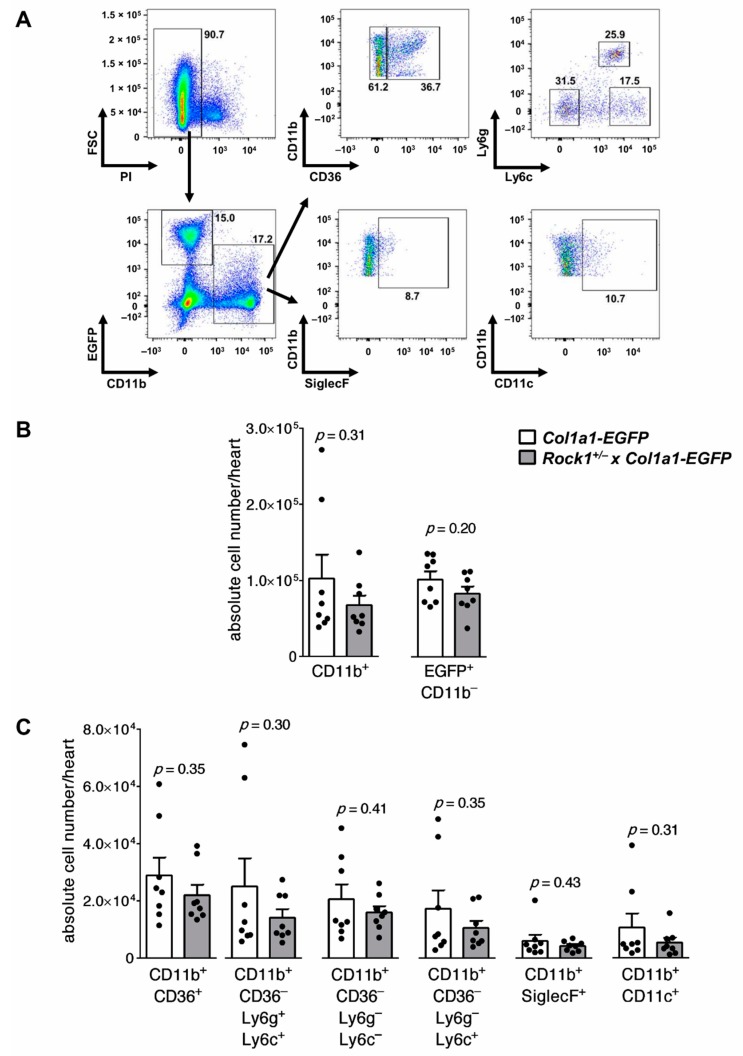
Flow cytometry analysis of cardiac inflammatory cells in wild-type and *Rock1* ^+/−^ mice at the acute stage of myocarditis. Panel (**A**) shows gating strategy for myeloid (CD11b^+^) cell subsets and cardiac fibroblasts (EGFP^+^CD11b^–^) in flow cytometry analysis of hearts obtained from mice with the *Coll-EGFP* reporter transgene. Analysis of unstained heart sample is shown in Supplementary Figure S2. Quantifications of total number of myeloid and cardiac fibroblasts, as well as indicated myeloid cell subsets in hearts of *Coll-EGFP* (n = 8) and *Rock1* ^+/−^ x *Coll-EGFP* (n = 8) mice, at day 21 of EAM are shown in panels (**B**) and (**C**), respectively. Each dot represents data for one heart, and bars present mean value ± SEM. *p*-value was calculated with the unpaired Student’s *t*-test.

In the next step, we analysed whether *Rock1* haploinsufficiency affected the number of cardiac fibroblasts or inflammatory myeloid cell subsets during the acute phase of EAM. For this purpose, we used a reporter mouse strain expressing EGFP under collagen type I promoter (*Coll-EGFP*), in which EGFP^hi^ cells indicated cardiac fibroblasts [27,32]. The flow cytometry analysis of hearts obtained from *Coll-EGFP* and *Rock1*^+/−^ x *Coll-EGFP* mice at day 21 of EAM showed a similar number of cardiac fibroblasts in both groups (Figure 2A,B). The total numbers of inflammatory CD11b^+^ myeloid cells were comparable in both groups (Figure 2B), which is in line with histological data. Further, we analysed myeloid cell subsets on gated cardiac CD11b^+^ cells. We observed no significant difference in total number (or percentage) of CD11b^+^CD36^+^ macrophages, CD11b^+^CD36^–^Ly6G^hi^Ly6c^hi^ neutrophils, CD11b^+^CD36^–^Ly6G^–^Ly6c^hi^ inflammatory monocytes, CD11b^+^CD36^–^Ly6G^–^Ly6c^–^ monocytes, CD11b^+^SiglecF^+^ eosinophils and CD11b^+^CD11c^+^ inflammatory dendritic cells between hearts of *Coll-EGFP* and *Rock1*^+/−^ x *Coll-EGFP* mice at day 21 of EAM (Figure 2C, Supplementary Table S1). These data suggest that ROCKs are not critically involved in the development of autoimmune myocarditis.

### 3.2. TGF-β Induces Profibrotic Changes in Cardiac Fibroblasts and in Inflammatory Myeloid Cells

**Figure 3:**
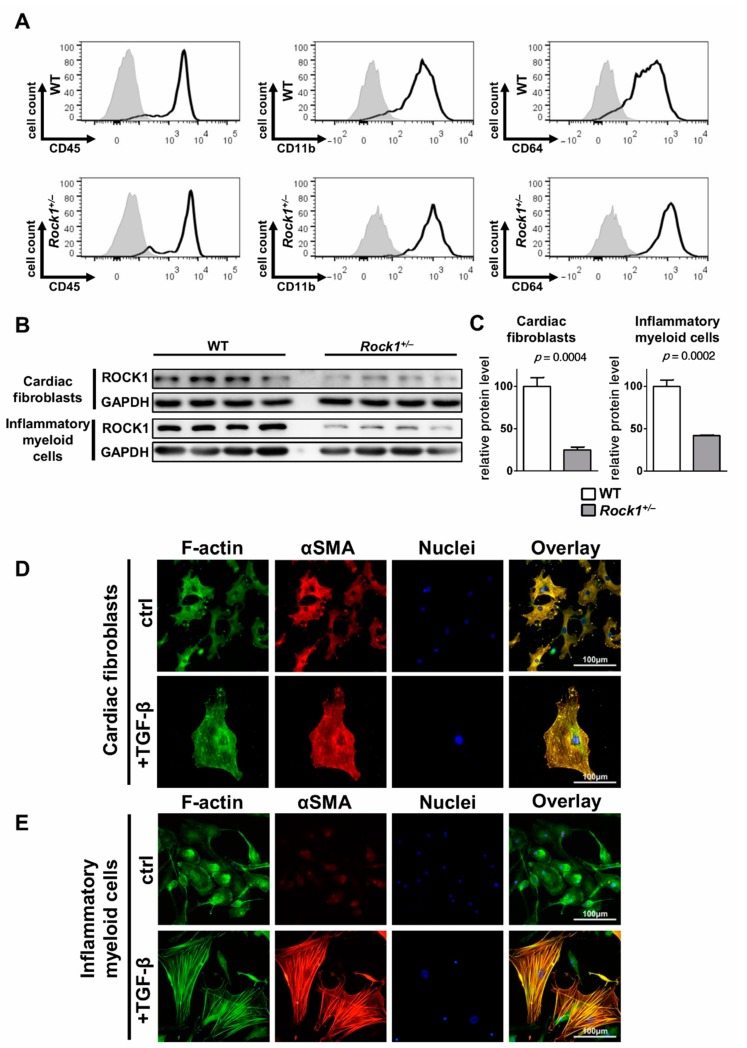
**TGF-β converts cardiac fibroblasts and inflammatory myeloid cells into myofibroblasts.** Panel (**A**) shows representative flow cytometry analysis of the indicated antigens for inflammatory myeloid cells isolated from wild-type or *Rock1*^+/−^ hearts at day 18-21 of EAM and expanded in vitro. Panel (**B**) shows immunoblots and panel (**C**) the respective quantifications of ROCK1 protein levels (normalized to GAPDH) in wild-type and *Rock1*^+/−^ cardiac fibroblasts and inflammatory myeloid cells. Panels (**D**) and (**E**) show representative immunofluorescence for αSMA (red) and F-actin (phalloidin staining, green) of cardiac fibroblasts (**A**) and inflammatory myeloid cells (**B**) cultured in the absence (ctrl) or presence of TGF-β (+TGF-β) for 3 days. Bars represent mean value ± SEM. n = 4, *p*-value was calculated with the unpaired Student’s *t*-test.

Profibrotic changes in the heart are typically associated with accumulation of αSMA-positive myofibroblasts. Resident cardiac fibroblasts represent a natural source of pathogenic myofibroblasts in the cardiac tissue. Previously, we have shown that in the EAM model, inflammatory myeloid cells (referred to earlier as prominin-1^+^/CD133^+^ progenitors [8,29]) could serve as an alternate source for myofibroblast-like cells in postinflammatory fibrosis [8]. Here, we expanded inflammatory myeloid cells from myocarditis hearts of wild-type and *Rock1*^+/−^ mice and showed that both groups were positive for myeloid markers CD45, CD11b and CD64 in flow cytometry analysis (Figure 3A). Next, we confirmed substantially reduced ROCK1 protein levels in *Rock1*^+/−^ haploinsufficient cardiac fibroblasts and inflammatory myeloid cells (Figure 3B,C). To study profibrotic changes, we used cardiac fibroblasts and inflammatory myeloid cells and treated them with TGF-β. Treatment with TGF-β for 3 days induced formation of αSMA-positive myofibroblasts in both cell types (Figure 3D,E); therefore, we decided to use both cell types to study the involvement of ROCK1 in TGF-β-induced fibrosis.

### 3.3. ROCK1 Differentially Regulates TGF-β Downstream Molecular Pathways in Cardiac Fibroblasts and in Inflammatory Myeloid Cells

**Figure 4:**
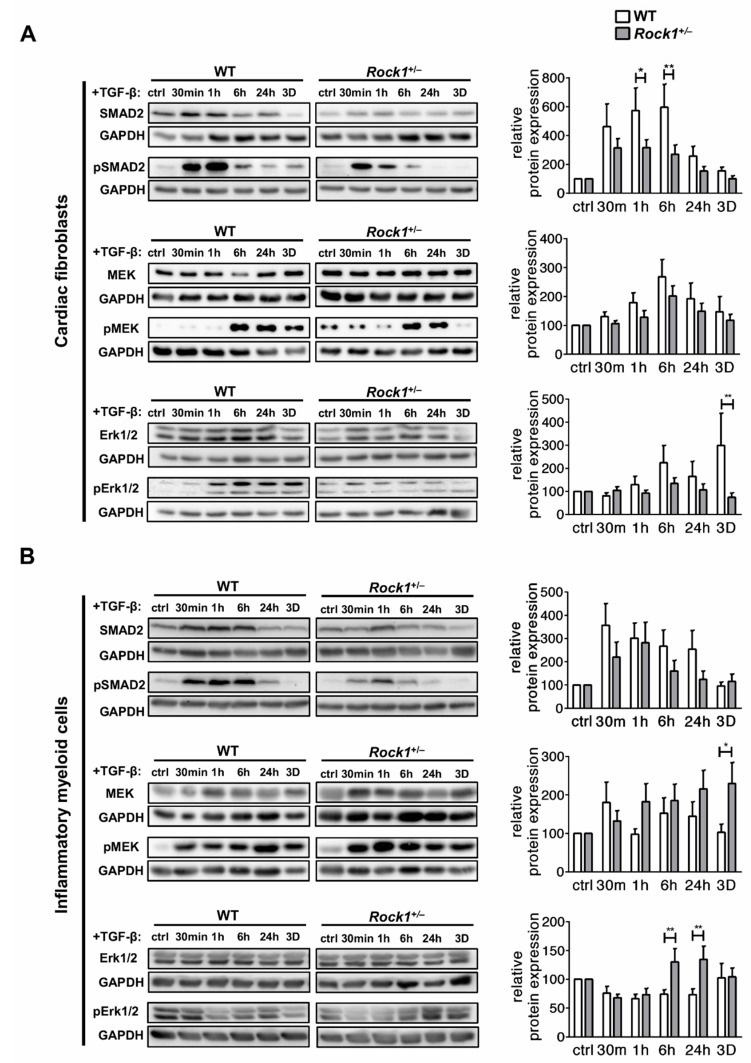
Differential molecular response of wild-type and *Rock1*^+/−^ cardiac fibroblasts and inflammatory myeloid cells to TGF-β Representative immunoblots and the respective quantifications of Smad2 activation (pSMAD2/SMAD2), MEK activation (pMEK/MEK) and Erk activation (pErk/Erk) in wild-type and *Rock1*^+/−^ cardiac fibroblasts (**A**) and inflammatory myeloid cells (**B**) at the indicated time point following TGF-β stimulation. Protein levels were normalized to GAPDH. Relative response to TGF-β was calculated in relation to internal untreated controls (ctrl) for wild-type and *Rock1*^+/−^ cells. Bars represent mean value ± SEM. n = 6–7, * *p* < 0.05, ** *p* < 0.01 calculated with the unpaired Student’s *t*-test for each time point.

In the next step, we analysed activation of two TGF-β downstream pathways: canonical Smad-dependent and noncanonical MEK/Erk in wild-type and *Rock1*^+/−^ cardiac fibroblasts and inflammatory myeloid cells. Treatment with TGF-β resulted in rapid (30 min) phosphorylation of Smad2 and activation of MEK/Erk at later time points. *Rock1*^+/−^ cardiac fibroblasts showed an impaired phosphorylation of Smad2 protein in response to TGF-β (Figure 4A). Furthermore, we observed unaffected short-term MEK/Erk response, but a long-term Erk phosphorylation was reduced in these cells (Figure 4A). On the other hand, treatment of wild-type inflammatory myeloid cells with TGF-β resulted in a potent Smad2 response, but a poor activation of MEK/Erk pathway. *Rock1*^+/−^ inflammatory myeloid cells showed a similar Smad2 phosphorylation response to TGF-β as wild-type cells, but an increased phosphorylation of MEK and Erk (Figure 4B). These data pointed to cell-type-specific involvement of ROCK1 in the molecular TGF-β response.

### 3.4. ROCK1 Enhances Profibrotic TGF-β Response in Cardiac Fibroblasts and in Inflammatory Myeloid Cells

**Figure 5:**
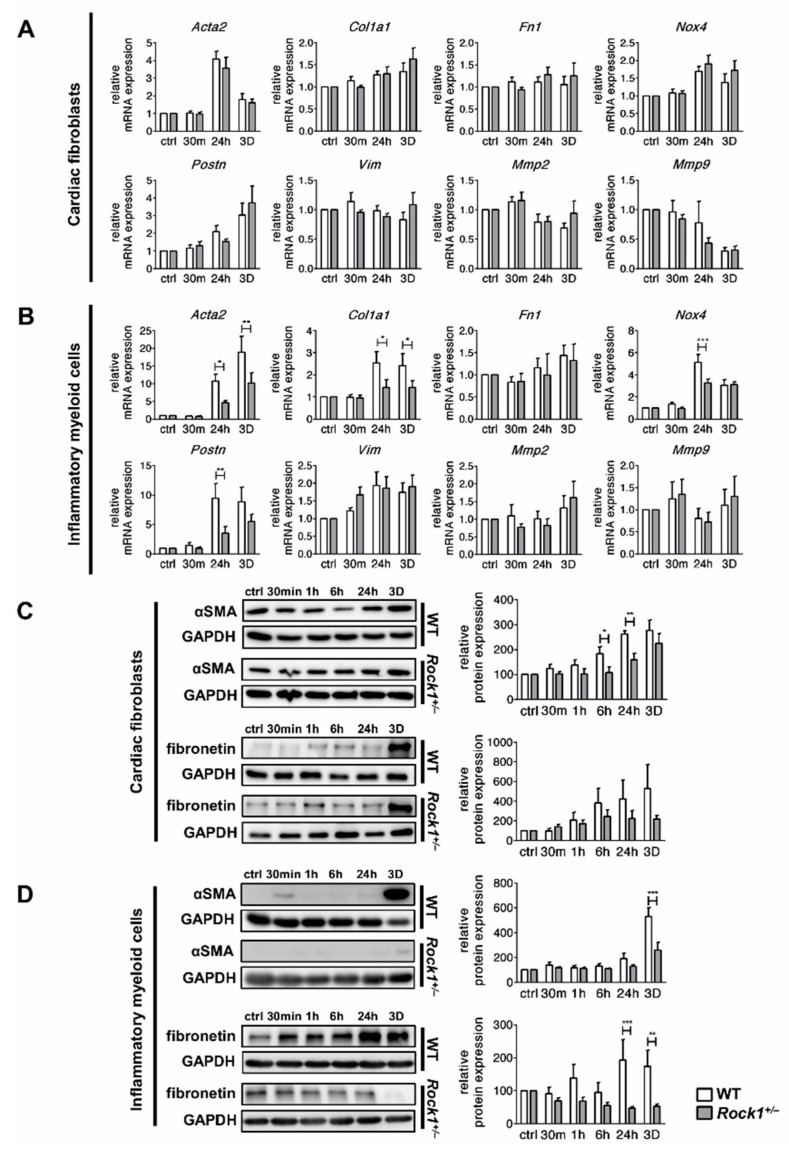
**ROCK1 differentially regulates profibrotic products in cardiac fibroblasts and in inflammatory myeloid cells.** Panels (**A**) and (**B**) show relative expression of selected profibrotic genes in wild-type and *Rock1*^+/−^ cardiac fibroblasts (**A**) (n = 6–8), and inflammatory myeloid cells (**B**) (n = 6–8), at the indicated time point following TGF-β stimulation. Gene expression was normalized to *Gapdh*. Panels (**C**) and (**D**) show representative immunoblots and the respective quantifications (normalized to GAPDH) of αSMA and fibronectin in wild-type and *Rock1*^+/−^ cardiac fibroblasts (C) (n = 7), and inflammatory myeloid cells (**D**) (n = 6–9), at the indicated time point following TGF-β stimulation. Relative response to TGF-β was calculated in relation to internal untreated controls (ctrl) for wild-type and *Rock1*^+/−^ cells. Bars represent mean value ± SEM. * *p* <0.05, ** *p* <0.01, *** *p* <0.001 calculated with the unpaired Student’s *t*-test for each time point.

Treatment with TGF-β induced profibrotic changes in cardiac fibroblasts and in inflammatory myeloid cells. Transcriptional response of profibrotic genes to TGF-β was similar in wild-type and *Rock1*^+/−^ cardiac fibroblasts (Figure 5A). In contrast, *Rock1*^+/−^ inflammatory myeloid cells showed impaired upregulation of several profibrotic genes, such as *Acta2*, *Col1a1*, *Nox4* and *Postn* (Figure 5B). Next, we analysed production of αSMA and fibronectin at protein levels. We observed that both profibrotic proteins showed reduced upregulation in *Rock1*^+/−^ cells (Figure 5C,D). These data could confirm ROCK1 involvement in profibrotic processes.

### 3.5. Rock1^+/−^ and Rock2^+/−^ Haploinsufficient Mice Are Not Protected from Cardiac Fibrosis in EAM

**Figure 6:**
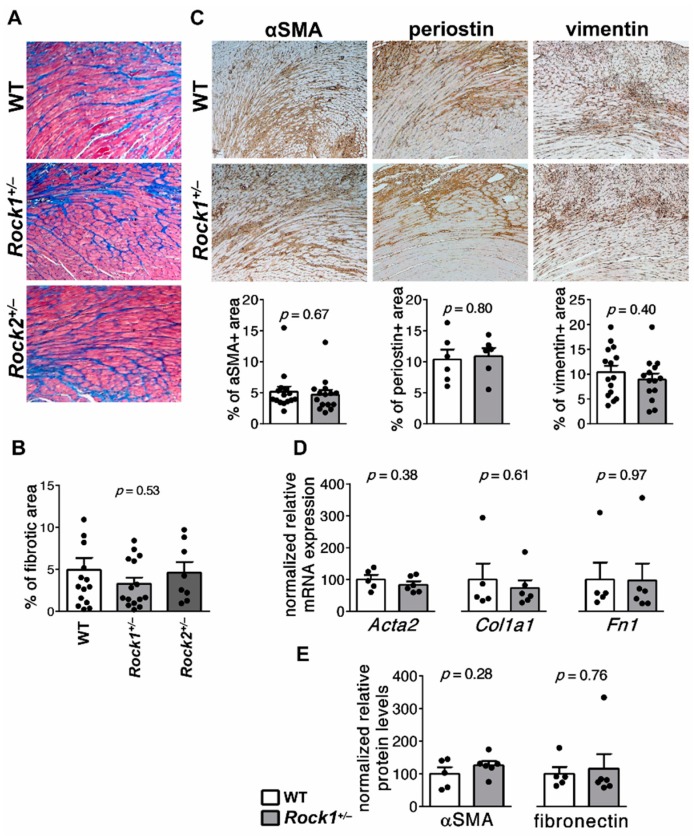
***Rock1***^+/−^**and*Rock2***^+/−^**mice are not protected from postinflammatory cardiac fibrosis.** Panel (**A**) shows representative Masson’s trichrome staining (indicating fibrosis) of heart tissue sections obtained from wild-type (n = 14), *Rock1*^+/−^ (n = 15) and *Rock2*^+/−^ (n = 8) mice at day 40 of EAM. Magnification ×400. Quantification of fibrotic area is shown in (**B**). Representative immunohistochemistry stainings for αSMA, periostin and vimentin on heart sections of wild-type and *Rock1*^+/−^ mice at day 40 of EAM and the respective quantifications of immunopositive areas are presented in (**C**). Panel (**D**) shows relative expression of selected profibrotic genes (n = 5) and panel (**E**) αSMA and fibronectin protein levels (n = 5) measured by immunoblotting in wild-type and *Rock1*^+/−^ hearts at day 40 of EAM. Each dot represents data for one mouse and bars present mean value ± SEM. *P*-values were calculated with the ANOVA test (B) or the unpaired Student’s *t*-test (**C**–**E**).

In the EAM model, acute inflammation of the myocardium is replaced by progressive cardiac fibrosis. At day 40 of EAM, most of the immunized mice developed patchy cardiac fibrosis. Unexpectedly, we found that the extent of cardiac fibrosis indicated by Masson’s trichrome staining was comparable in *Rock1*^+/−^ and wild-type mice (Figure 6A,B). Similarly, *Rock2*^+/−^ haploinsufficient mice also developed unaffected cardiac fibrosis at day 40 of EAM (Figure 6A,B). Next, we took fibrotic hearts of *Rock1*^+/−^ and wild-type mice and analysed them by immunohistochemistry for the presence of vimentin as a marker of fibroblasts, and αSMA and periostin as indicators of activated fibroblasts and myofibroblasts. We detected all three proteins in the interstitial space of the cardiac tissues and found no significant differences between *Rock1*^+/−^ and wild-type groups (Figure 6C). No differences in the profibrotic products between *Rock1*^+/−^ and wild-type hearts were further confirmed at transcriptional (*Acta2*, *Col1a1*, *Fn1*, Figure 6D) and protein (αSMA and fibronectin, Figure 6E) levels. These data suggest that ROCKs are not critically involved in the development of postinflammatory fibrosis in the heart.

## 4. Discussion

ROCKs have been associated with fibrotic processes in the heart [33]. Data from experimental animal models showed that complete or partial deletion of *Rock1* or *Rock2* protected mice from fibrosis during hypertensive cardiac remodelling [16,21,26] and following myocardial infarction [16]. Furthermore, cardioprotective, antifibrotic effects were observed by treatment with pharmacological ROCK inhibitors [19,34,35,36]. Insight from other models further pointed to profibrotic role of ROCKs in pulmonary fibrosis [37] and in diabetic kidney injury [38]. In light of these data, our findings showed unexpectedly that *Rock1*^+/−^ and *Rock2*^+/−^ were not protected from the development of postinflammatory fibrosis in mouse model of EAM. 

It should be noted that in EAM, acute inflammation is a primary trigger of tissue remodelling and cardiac fibrosis. In contrast, during hypertensive cardiac remodelling, the typical inflammatory cells are virtually not present in the myocardium. On the other hand, during early phase of myocardial infarction, there is a certain degree of inflammation in the affected region, but the response to ischemia is a main mechanism that induces profibrotic activity in cardiac fibroblasts. Recent data demonstrate that local sterile inflammation is, in fact, beneficial for the infarcted heart [39]. Obviously, molecular triggers differ between different cardiac conditions. We think that involvement of ROCKs in fibrosis might critically depend on the extent of activation of the specific molecular pathways, such as G protein-coupled receptor signalling, and/or rely on significant involvement of a specific cell type, such as activated cardiomyocytes. A facultative profibrotic involvement of ROCKs has already been observed in the kidney. *Rock1*^−/−^ mice showed attenuated progression of renal fibrosis during streptozotocin-induced diabetic injury [38], whereas in the unilateral ureteral obstruction model, *Rock1*^−/−^ mice developed unaffected fibrosis in the kidney [40].

Rho-ROCKs represent direct signal transduction pathways of the G protein-coupled receptor signalling [41]. It is therefore not surprising that critical involvement of ROCKs in fibrotic processes was observed in models induced by constitutive overexpression of Gαq proteins [14]. Similar potent activation of Rho-ROCKs pathways occurs in hypertensive model induced by angiotensin II infusion, as angiotensin II receptors represent a type of G protein-coupled receptors [42]. In these models, disease-inducing factors directly act on ROCKs. In the EAM model, on the other hand, fibrotic processes are orchestrated by multiple molecular agents, such as TGF-β, Wnts, IL-1 or IL-17A, that activate a number of profibrotic pathways [8,43,44,45]. It seems that inflammation is, in general, a weak activator of ROCK pathways in the heart. It is possible that other cardiac pathogenic conditions, like infarction and hypertension, more potently activate ROCKs.

TGF-β represents one of the most important profibrotic cytokines that play a critical role in the development of postinflammatory fibrosis in EAM [6]. Our in vitro data showed that ROCK1 maintained the canonical Smad2 response in TGF-β-activated cardiac fibroblasts. These data are in line with previous reports showing that blockade of RhoA or ROCK effectively reduced TGF-β-mediated activation of Smad signalling [46,47]. Furthermore, in our model, the stimulation of *Rock1*^+/−^ cardiac fibroblasts with TGF-β resulted in reduced Erk phosphorylation. Impaired Erk activation has been also observed in TGF-β-activated cardiac fibroblasts treated with ROCK inhibitor ZYZ-168 [20] and in postinfarcted hearts of rats receiving ROCK inhibitor fasudil [19]. These data suggested that ROCKs could enhance canonical and noncanonical TGF-β downstream molecular responses, and a positive feedback loop mechanism could serve as a potential explanation. Indeed, activation of Rho-ROCKs has been linked with TGF-β production [16,21,38] supporting such a regulatory mechanism. It should be noted that the postulated positive feedback mechanism is limited to specific cell types or specific pathogenic condition. In our experiments, ROCK1 failed to regulate Smad2 phosphorylation in TGF-β-treated inflammatory myeloid cells and acted in these cells as a negative regulator of Erk. We can speculate that the differential involvement of ROCK1 in TGF-β signalling between cardiac fibroblasts and inflammatory myeloid cells occurred due to potential differences in endogenous TGF-β production or in activation of TGF-β-related pathways between these cell types. Furthermore, a negative regulation of TGF-β production and activation of canonical Smad pathway have been observed in kidneys of *Rock1*^−/−^ mice following the unilateral ureteral obstruction [40].

In the heart, ROCKs have been studied mainly in the context of hypertrophy and cardiac fibrosis. In this study, we also addressed their contribution also to the development of autoimmune myocarditis and found that *Rock1*^+/−^ and *Rock2*^+/−^ developed unaffected CD4^+^ T cell-mediated cardiac inflammation. Insight from other models suggested, however, the importance of the Rho-ROCK pathway in autoimmunity. For example, genetic deletion of *Rhoa* in T cells reduced severity of experimental autoimmune encephalomyelitis [48]. A similar neuroprotective effect was observed by blocking ROCK activity with fasudil [49]. Furthermore, fasudil effectively ameliorated development of spontaneous arthritis and systemic lupus erythematosus in transgenic mouse models [50]. Of note, in viral myocarditis model, mice treated with fasudil showed reduced coxsackievirus B3 replication and attenuated myocardial necrotic lesions [51]. Noteworthy, in contrast to numerous studies testing pharmacological ROCK inhibitors in autoinflammatory models, studies using ROCK genetic deficiencies are limited. In the mouse model of asthma, *Rock1*^+/−^ and *Rock2*^+/−^ mice showed attenuated mast cell degranulation and reduced number of eosinophils, but both developed unaffected lung inflammation [52].

Compounds targeting Rho-ROCK pathways such as fasudil are therapeutically valuable and have been successfully used in the clinic for many years. It should be noted, however, that beneficial effects of pharmacological ROCK inhibitors might be at least partly mediated by targeting off-target kinases [53]. In order to fully understand molecular pathomechanisms of the diseases, experimental data with highly specific targeting of individual components of the Rho-ROCK pathways are needed. In the case of addressing the roles of ROCKs in animal models, there are two important limitations. Many mouse models are available in mice on C57BL/6 or BALB/c genetic background. Constitutive *Rock1*^−/−^ and *Rock2*^−/−^ homozygotes are viable predominantly on FvB background [15,21,38,40]. Previous study reported that *Rock1*^−/−^ and *Rock2*^−/−^ homozygotes die prenatally on C57BL/6 background [16]. Here, we showed a similar strong underrepresentation of *Rock1*^−/−^ and *Rock2*^−/−^ homozygotes on BALB/c background. The use of the Cre-flox system represents an attractive alternative for the constitutive genetic deletion. In fact, this system has been successfully used to knock-down *Rock2* in cardiomyocytes [23]. Furthermore, it should be mentioned that ROCK1 and ROCK2 isoforms share strong homology. Both isoforms are often involved in the same processes, although they also possess nonredundant functions [13]. In specific cases, loss of one isoform could be compensated for by the activity of the other one. Thus, phenotypical changes observed in *Rock1*^+/−^ or *Rock2*^+/−^ haploinsufficient mice should point to rather strong involvement of ROCKs.

In summary, our data suggest that neither ROCK1 nor ROCK2 is critically involved in the development of CD4^+^ T cell-mediated myocarditis and postinflammatory fibrosis. We cannot, however, exclude that the Rho-ROCK pathway is redundant in this model. Furthermore, our data could confirm the regulation of the downstream TGF-β molecular response by ROCK1, but also highlighted marked differences between cell types in this mechanism.

## Supplementary Materials

The following are available online at https://www.mdpi.com/2073-4409/9/3/700/s1, Figure S1: Genotype of *Rock1*^+/−^ and *Rock2*^+/−^ offspring, Figure S2: Flow cytometry analysis of unstained mouse heart Table S1: Oligonucleotide sequences used in quantitative RT-PCR, Table S2: Quantification of cardiac inflammatory cells in *Coll-EGFP* and *Coll-EGFP* x *Rock1*^+/−^ mice at d21 of EAM.
