# Supplementary material for: Haploinsufficient Rock1+/− and Rock2+/− Mice Are Not Protected from Cardiac Inflammation and Postinflammatory Fibrosis in Experimental Autoimmune Myocarditis
Source: Cells. 2020 Mar 12;9(3):700. doi: 10.3390/cells9030700 (PMC7140701; doi:10.3390/cells9030700)
Supplement: Supplementary file 1 [file cells-09-00700-s001.pdf]

Supplementary materials

A

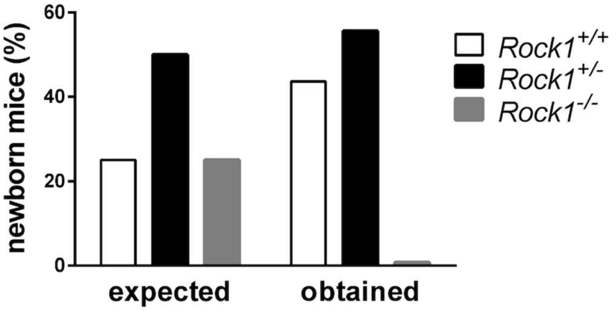

B

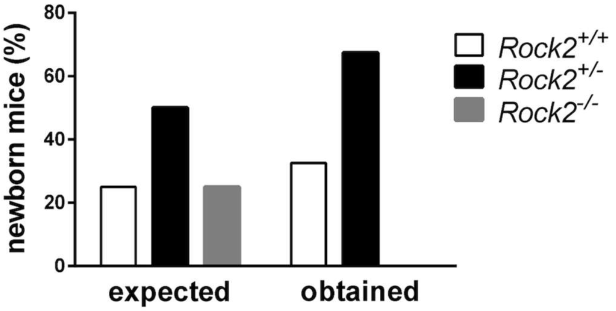

Figure 1. Genotype of *Rock1*$^{+/-}$ and *Rock2*$^{+/-}$ offspring. Percentages of expected (left) and actual (right) genotypes of newborn mice obtained from haploinsufficient breedings of *Rock1*$^{+/-}$ x *Rock1*$^{+/-}$ (n=252, A) and *Rock2*$^{+/-}$ x *Rock2*$^{+/-}$ (n=227, B).

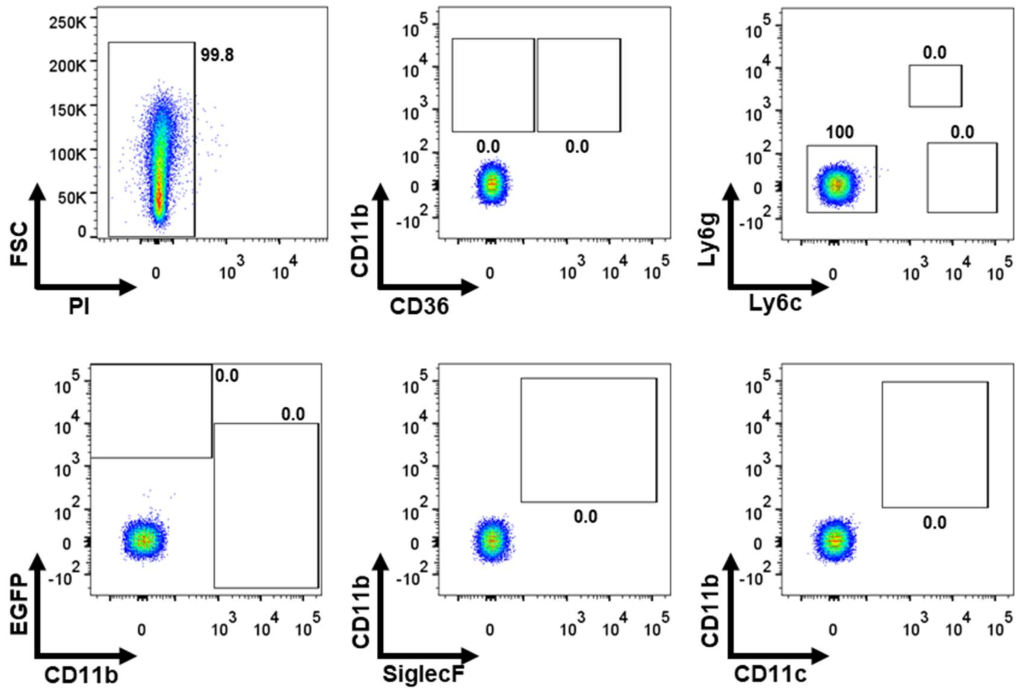

Figure 2. Flow cytometry analysis of unstained mouse heart. Gating strategy for myeloid cell subsets and cardiac fibroblasts in flow cytometry analysis of unstained samples of heart from wild-type mice.

Table 1. Oligonucleotide sequences used in quantitative RT-PCR. Forward and reverse sequences of oligonucleotides complementary to transcripts of the analyzed genes used in quantitative RT-PCR experiments.

Gene	F primer sequence	R primer sequence
Acta2	GCCATCTTTCATTGGGATGGAG	ACAGGACGTTGTTAGCATAGAGA
Col1a1	ACCGATGGATTCCCGTTCG	CATTAGGCGCAGGAAGGTCA
Fn1	GAACGACACGTTCCACAAGC	CACTGGTCAATGGGGTCACA
Nox4	TGGCCAACGAAGGGGTTAAA	ACACAATCCTAGGCCCAACA
Postn	AGACTGCTTCAGGGAGACAC	ACGGCCTTCTCTTGATCGTC
Vim	CGAGAGAAATTGCAGGAGGAGA	CGTTCAAGGTCAAGACGTGC
Mmp2	CCCCCATGAAGCCTTGTTTAC	TACAGCTGTTGTAGGAGGTGC
Mmp9	AGCGCCAGCCGACTTTT	TAGCGGTACAAGTATGCCTCTG
Rock1	AAGCTTGCAGGAAAGTTTACAGA	AAGTTCTGCATTTCTGCTCCA
Gapdh	GTCCCAGCTTAGGTTCATCAGG	ACGGCCAAATCCGTTCACAC

Table S2. Quantification of cardiac inflammatory cells in *Coll-EGFP* and *Coll-EGFP x Rock1*$^{+/-}$ mice at d21 of EAM. Table presents percentages and absolute numbers (mean value ±SEM) of analyzed cells populations in *Coll-EGFP* and *Coll-EGFP x Rock1*$^{+/-}$ mice hearts at day 21 of EAM. EGFP^{+} cardiac fibroblasts and inflammatory CD11b^{+} myeloid cells were analyzed on total single cells (total EGFP^{+} and total CD11b^{+}) and on myeloid cell subsets gated on inflammatory CD11b^{+} myeloid cells as presented in the Figure 2.

	**Coll-EGFP**		**Coll-EGFP x ROCK1$^{+/-}$**	
	**Percentage**	**Absolute number**	**Percentage**	**Absolute number**
**Total EGFP^{+}**	16.75 (±1.90)	160227 (±31014)	14.58 (±1.55)	113528 (±17536)
**Total CD11b^{+}**	8.74 (±1.48)	102551 (±30873)	7.90 (±1.05)	67604 (±12209)
**CD11b^{+}-gated cells**				
**CD11b^{+} CD36^{+}**	34.81 (±6.30)	288789 (±6146)	35.53 (±5.68)	21948 (±3553)
**CD11b^{+} CD36^{-} Ly6g^{+} Ly6c^{+}**	21.28 (±2.49)	25045 (±9719)	20.21 (±1.24)	14148 (±2910)
**CD11b^{+} CD36^{-} Ly6g^{-} Ly6c^{-}**	22.81 (±3.20)	20630 (±50870)	26.21 (±4.01)	15936 (±2146)
**CD11b^{+} CD36^{-} Ly6g^{-} Ly6c^{+}**	15.07 (±2.23)	17216 (±6399)	14.97 (±1.60)	10528 (±2457)
**CD11b^{+} SiglecF^{+}**	6.68 (±1.16)	5970 (±2110)	7.18 (±1.16)	4190 (±632)
**CD11b^{+} CD11c^{+}**	9.84 (±1.26)	10671 (±4775)	9.57 (±1.67)	5361 (±1645)
